# Prognostic Utility of the Preoperative Cachexia Index in Patients Undergoing Emergency Laparotomy

**DOI:** 10.1002/ags3.70097

**Published:** 2025-10-06

**Authors:** Naoko Fukushima, Takahiro Masuda, Kazuto Tsuboi, Masami Yuda, Keita Takahashi, Fumiaki Yano, Ken Eto

**Affiliations:** ^1^ Department of Surgery The Jikei University School of Medicine Minato‐ku, Tokyo Japan; ^2^ Department of Surgery Fuji City General Hospital Fuji‐shi, Shizuoka Japan

**Keywords:** cachexia index, emergency laparotomy, gastric cancer, neutrophil‐to‐lymphocyte ratio, sarcopenia

## Abstract

**Aim:**

Emergency laparotomy is associated with high morbidity and mortality rates. The cachexia index, an objective index that incorporates muscle mass, nutrition, and inflammation, has been shown to predict outcomes in patients with cancer. The present study aimed to investigate the prognostic significance of preoperative cachexia index in patients undergoing emergency laparotomy.

**Methods:**

This retrospective study included data from 404 patients who underwent emergency laparotomy between January 2013 and March 2023. The cachexia index was calculated as: (psoas muscle index × albumin level [g/dL]/neutrophil‐to‐lymphocyte ratio). Patients were divided into low and high cachexia index groups based on sex‐specific receiver operating characteristic curve derived cut‐off values.

**Results:**

Of 404 patients (median age, 74 years [44.6% female]), 120 (30%) exhibited a low cachexia index. In‐hospital mortality was significantly higher in the low cachexia index group (20.0%) than that in the high cachexia index group (5.0%) (*p* < 0.01). Multivariate analysis revealed that age, chronic renal failure and cachexia index were independent predictors of in‐hospital mortality. One‐year mortality was also higher in the low versus high cachexia index group (34.2% vs. 13.4%, respectively; *p* < 0.01).

**Conclusion:**

Preoperative cachexia index was a useful marker for predicting mortality after emergency laparotomy and may aid in risk stratification and perioperative decision‐making.

## Graphical abstract

The preoperative cachexia index, incorporating muscle mass, nutritional status, and inflammation, predicts in‐hospital and one‐year mortality in patients undergoing emergency laparotomy. This simple index may aid in risk stratification and perioperative decision‐making in high‐risk surgical patients.

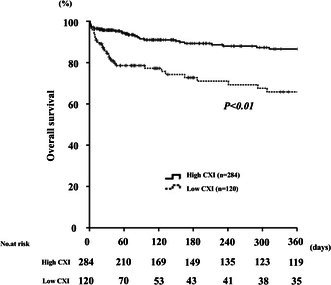

## 1 Introduction

Emergency laparotomy (EL) has one of the highest complication and mortality rates among all types of surgery. Medical advances and developments have led to gradual decreases in the 30‐day mortality rate for EL, from 11.8% in 2013 to 9.6% in 2018 [1, 2]; nevertheless, it remains high. The 1‐year mortality rate has been reported to be 24.6% [1, 2]. Assessment of patient‐specific risk factors for postoperative mortality is critical for clinical decision‐making and for improving patient outcomes. Many scoring systems, such as the National Emergency Laparotomy Audit (NELA) [3], Portsmouth physiological and Operative Severity Score for the Enumeration of Mortality and Morbidity (P‐POSSUM) [4], Acute Physiology and Chronic Health Evaluation II (APACHE II) score [5] have been developed to estimate the prognosis of critically ill patients. However, these scoring systems require numerous variables, which can be challenging to assess. Additionally, these measures do not adequately capture patient general health status before admission.

Sarcopenia has recently been recognized as an independent predictor of postoperative mortality in patients who undergo EL [6]. It has also been reported that the neutrophil‐to‐lymphocyte ratio (NLR) and albumin (Alb) level obtained from preoperative laboratory investigations are useful prognostic predictors [7, 8]. The cachexia index (CXI) is a novel biomarker comprising the skeletal muscle index (SMI), serum Alb, and NLR, and the association between poor prognosis and low CXI has been reported in patients with various cancers [9]. The CXI is an index that combines sarcopenia, which reflects patient condition before admission, with NLR and Alb. Therefore, the CXI is considered to reflect both patient condition before admission and current status before surgery, suggesting its potential utility as a preoperative indicator. However, the utility of preoperative CXI for predicting the prognosis of patients undergoing EL has not yet been reported. As such, this study aimed to clarify the prognostic utility of preoperative CXI in patients who underwent EL, with particular focus on in‐hospital mortality and overall survival.

## 2 Methods

### 2.1 Study Design

This study included all consecutive patients who underwent EL at Fuji City General Hospital (Shizuoka, Japan) between January 2013 and March 2023. Initially, 1261 patients were identified. Those younger than 18 years (*n* = 184), those with appendicitis (*n* = 480), cholecystitis (*n* = 66), inguinal hernia without bowel resection (*n* = 105), or trauma (*n* = 22) were excluded, leaving data from 404 patients included in the analysis. These exclusion criteria were based on the criteria established by the United Kingdom (UK) NELA Collaborative Study [3]. A prospectively maintained patient database was retrospectively reviewed. Variables collected from the database included age, sex, body mass index (BMI), American Society of Anesthesiologists (ASA) classification [10], patient comorbidities, operative data, and postoperative course. In addition, preoperative laboratory data and computed tomography (CT) within 1 day of surgery were also collected. Postoperative complications were defined as grade ≥ III according to the Clavien–Dindo classification [11]. The P‐POSSUM system incorporates two categories of scoring: the physiology score (PS) and the operative severity score (OS). The PS is derived from 12 clinical and laboratory variables, including age, cardiac status, respiratory history, systolic blood pressure, pulse rate, Glasgow Coma Scale score, hemoglobin level, white blood cell count, plasma urea, plasma sodium, plasma potassium, and electrocardiographic findings. In contrast, the OS is determined using six operative factors: operative severity, the performance of multiple procedures, total blood loss, degree of peritoneal soiling, presence of malignancy, and mode of surgery. The P‐POSSUM predicted mortality rate (R) was calculated as follows: lnR/(1 − R) = −9.065 + 0.1692 × PS + 0.1550 × OS [4]. This retrospective study was approved by the Ethics Committee of the Fuji City General Hospital (No. 296).

### 2.2 Definition of CXI

**FIGURE 1:**
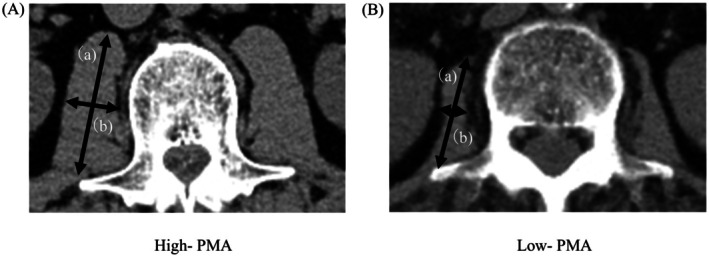
Measurement of the psoas muscle area (PMA) at the level of the third lumbar vertebra. The PMA was calculated using the following formula: Major axis length (a) × minor axis length (b) × π. (A) High PMA, (B) low PMA.

The CXI was calculated as follows: (SMI × serum Alb [g/dL]/NLR) [9]. The psoas muscle index (PMI) was used as a substitute for SMI due to the complexity of measuring this parameter. Psoas muscle area (PMA) was calculated by measuring the major and minor diameters of the psoas muscle at the third lumbar vertebra (L3) using preoperative CT [12]. PMA was calculated as follows: length of the major axis of the psoas muscle (cm) × minor axis (cm) × π (Figure 1). The PMI was then derived by normalizing the PMA to patient height squared (cm^2^/m^2^). Automated body composition software was not available during the early years of the study period, and to maintain methodological consistency across the 10‐year dataset, manual measurement was performed. In addition, the measurements were performed by two gastrointestinal surgeons (N.F. and K.T.) experienced in CT image analysis, with both evaluators blinded to the patients' clinical outcomes during the assessments. NLR was calculated by dividing the absolute neutrophil count by the absolute lymphocyte count [13]. The optimal cut‐off value for the CXI was determined using receiver operating characteristic (ROC) analysis of data stratified according to sex.

### 2.3 Analyses of Risk Factors for In‐Hospital Mortality and Survival

The primary outcome was in‐hospital mortality rate. Multivariate logistic regression analysis was performed to investigate the association between clinicopathological variables and in‐hospital mortality after EL. In addition, overall survival (OS) was analyzed using the Cox proportional hazards model. Pre‐specified prognostic variables included age, sex, Alb, CXI, PMI, NLR, malignancy, P‐POSSUM mortality rate, comorbidity, and postoperative diagnosis.

### 2.4 Statistical Analysis

Statistical analysis was performed using SPSS version 22.0 (IBM Corp. Inc., Armonk, NY, USA). Differences with *p* < 0.05 were considered to be statistically significant. Data are expressed as median, range, and ratio. The Mann–Whitney *U* and chi‐squared tests were used to compare continuous and dichotomous variables, respectively. Survival was estimated using Kaplan–Meier plots with a log‐rank test. Univariate and multivariate analyses were conducted to identify independent prognostic factors, using logistic regression for in‐hospital mortality and the Cox proportional hazards model for overall survival. Multivariate analysis was performed for factors with *p* < 0.05. The optimal cut‐off values for age, hemoglobin (Hb), creatinine (Cre), Alb, P‐POSSUM, and CXI were determined using ROC curve analysis, and the Youden Index was applied to identify the threshold that maximized sensitivity and specificity for in‐hospital mortality.

## 3 Results

### 3.1 Patient Characteristics

In total, 404 patients underwent EL, with baseline characteristics summarized in Table 1. The median age of the cohort was 74 years and 180 patients (44.6%) were female. Eighty‐five patients were ASA class ≥ 3 (21%). The optimal cut‐off value of CXI was 1.43 for males and 1.06 for females. The area under the curve for predicting in‐hospital mortality was 0.63 for males and 0.74 for females (Figure S1). Of the 404 patients, 120 (30%) were assigned to the low CXI group and 284 (70%) to the high CXI group. Patients with low CXI were significantly older (*p* < 0.01), had more comorbidities (cardiovascular disease; *p* = 0.04, chronic renal failure; *p* < 0.01, and cerebrovascular disease; *p* < 0.01), and a lower BMI (*p* < 0.01). Laboratory investigations revealed that patients with a low CXI exhibited significantly higher white blood cell (*p* < 0.01), Cre (*p* = 0.02), and C‐reactive protein levels (*p* < 0.01) and lower Hb (*p* < 0.01) and Alb (*p* < 0.01) levels.

**TABLE 1:** Patient characteristics.

Variables	Total (*n* = 404)	CXI		p
		Low (*n* = 120)	High (*n* = 284)	
Age, yeas	74 (59–82)	79 (68–83)	71 (56–80)	< 0.01
Gender, female	180 (44.6%)	61 (50.8%)	119 (41.9%)	0.10
Body mass index, kg/m^2^	21.0 (18.5–23.4)	19.9 (17.7–22.3)	21.4 (18.8–23.6)	< 0.01
Laboratory data				
WBC, /μL	10 700 (7700–15 200)	13 100 (9300–17 700)	10 000 (7000–14 500)	< 0.01
Hb, g/dL	13.2 (11.1–14.7)	11.9 (9.9–14.2)	13.5 (11.7–14.9)	< 0.01
Plt, ×10^4^/μL	23.1 (18.4–29.7)	23.0 (17.6–29.8)	23.3 (18.7–29.5)	0.79
Alb, g/dL	3.6 (2.9–4.1)	3.3 (2.3–3.8)	3.8 (3.1–4.2)	< 0.01
Bil, mg/dL	0.7 (0.5–1.0)	0.7 (0.5–1.0)	0.7 (0.5–1.0)	0.63
Cre, mg/dL	0.91 (0.69–1.32)	1.00 (0.72–1.78)	0.88 (0.68–1.20)	0.02
CRP, mg/dL	1.77 (0.19–14.38)	8.95 (1.53–21.6)	0.91 (0.1–9.1)	< 0.01
NLR	11.4 (6.4–20.7)	26.9 (16.4–36.6)	8.8 (4.9–13.7)	< 0.01
PMI, cm^2^				
Female	6.7 (4.5–9.2)	4.5 (3.0–6.2)	8.1 (5.8–10.4)	< 0.01
Male	9.7 (6.8–12.6)	7.1 (4.6–9.7)	10.5 (7.7–13.1)	< 0.01
ASA				
1	111 (27.5%)	26 (21.7%)	85 (29.9%)	< 0.01
2	208 (51.5%)	57 (47.5%)	151 (53.2%)	
≥ 3	85 (21.0%)	37 (30.8%)	48 (16.9%)	
Comorbidity				
Cardio vascular disease	54 (13.4%)	23 (19.2%)	31 (10.9%)	0.04
Respiratory disease	22 (5.5%)	6 (5.0%)	16 (5.6%)	0.82
Chronic renal failure	19 (4.7%)	12 (10.0%)	7 (2.5%)	< 0.01
Diabetes	52 (12.9%)	15 (12.5%)	37 (13.0%)	1.00
Cerebral vascular disease	37 (9.2%)	19 (15.8%)	18 (6.3%)	< 0.01
P‐POSSUM mortality rate, %	26.7 (17.8–41.1)	31.0 (21.5–45.2)	24.6 (15.7–38.7)	< 0.01

Abbreviations: Alb, albumin; ASA, American Society of Anesthesiologists; Bil, bilirubin; Cre, creatinine; CRP, C‐reactive protein; CXI, cachexia index; Hb, hemoglobin; NLR, neutrophil–lymphocyte ratio; Plt, platelet; PMI, psoas muscle index; P‐POSSUM, Portsmouth Physiological and Operative Severity Score for the en Umeration of Mortality and morbidity; WBC, white blood cell.

### 3.2 Surgical Outcome and Operative Course

Surgical outcomes and operative course are reported in Table 2. Postoperative diagnoses included gastrointestinal perforation (54.6%), small bowel obstruction (34.4%), large bowel obstruction (6.2%), ischemia (3.7%), and others (1.1%). There were no significant differences in postoperative diagnosis and malignancy, regardless of CXI (*p* = 0.06, *p* = 0.38, respectively). A total of 115 postoperative complications, defined as Clavien–Dindo grade ≥ III occurred in both groups (28.5%). The most frequent complication was sepsis (*n* = 33, 8.2%), followed by surgical site infection (*n* = 30, 7.4%), respiratory failure (*n* = 13, 3.2%), leakage (*n* = 12, 3.0%), and both abscess and ileus (*n* = 11, 2.7% each). Postoperative complications were more common and hospital stay was longer in the low CXI group (*p* < 0.01, *p* < 0.01, respectively).

**TABLE 2:** Surgical outcome and operative course.

Variables	Total (*n* = 404)	CXI		p
		Low (*n* = 120)	High (*n* = 284)	
Operative time, min	108 (76–140)	120 (82–148)	106 (73–137)	0.06
Blood loss, mL	5 (0–85)	10 (0–100)	5 (0–80)	< 0.01
Postoperative diagnosis				
Small bowel obstruction	139 (34.4%)	32 (26.7%)	107 (37.7%)	0.06
Large bowel obstruction	25 (6.2%)	9 (7.5%)	16 (5.6%)	
Gastrointestinal perforation				
Stomach and duodenum	81 (20.0%)	30 (25.0%)	51 (18.0%)	
Small intestine	24 (5.9%)	6 (5.0%)	18 (6.3%)	
Colon and rectum	116 (28.7%)	33 (27.5%)	83 (29.2%)	
Ischemia	15 (3.7%)	9 (7.5%)	6 (2.1%)	
Others	4 (1.1%)	1 (0.8%)	3 (1.1%)	
Malignancy, yes	43 (10.6%)	10 (8.3%)	33 (11.6%)	0.38
Postoperative complication (Clavien–Dindo Grade III–V)	115 (28.5%)	50 (41.7%)	65 (23.0%)	< 0.01
Sepsis	33 (8.2%)	20 (16.7%)	13 (4.6%)	
Surgical site infection	30 (7.4%)	6 (5.0%)	24 (8.5%)	
Respiratory failure	13 (3.2%)	9 (7.5%)	4 (1.4%)	
Leakage	12 (3.0%)	3 (2.5%)	9 (3.2%)	
Abscess	11 (2.7%)	5 (4.2%)	6 (2.1%)	
Ileus	11 (2.7%)	4 (3.3%)	7 (2.5%)	
Others	6 (1.5%)	3 (2.5%)	2 (0.7%)	
Reoperation	17 (4.3%)	4 (3.4%)	13 (4.6%)	0.61
In‐hospital mortality	38 (9.4%)	24 (20.0%)	14 (4.9%)	< 0.01
Overall mortality	93 (23.0%)	35 (29.2%)	58 (20.4%)	< 0.01
Sepsis	31 (7.7%)	19 (15.8%)	12 (4.2%)	
Cancer	25 (6.2%)	3 (2.5%)	22 (7.7%)	
Pneumonia	16 (4.0%)	8 (6.7%)	8 (2.8%)	
Cardiovascular disease	3 (0.7%)	0 (0%)	3 (1.1%)	
Gastrointestinal disease	3 (0.7%)	1 (0.8%)	2 (0.7%)	
Liver failure	2 (0.5%)	2 (1.7%)	0 (0%)	
Others	4 (1.0%)	0 (0%)	4 (1.4%)	
Uncertain	9 (2.2%)	2 (1.7%)	7 (2.5%)	
Postoperative hospital stay, days	17 (10–31)	18 (12–34)	16 (10–30)	< 0.01

Abbreviation: CXI, cachexia index.

### 3.3 Mortality

The in‐hospital mortality rate was 9.4% (*n* = 38), with a higher in‐hospital mortality rate in the low CXI group than that in the high CXI group (20% vs. 5%; *p* < 0.01). A summary of the univariate and multivariate analyses of clinicopathological variables associated with in‐hospital mortality after EL is presented in Table 3. In the univariate analysis, the significant prognostic factors for in‐hospital mortality included age ≥ 75 years (*p* < 0.01), Alb ≥ 3.3 g/dL (*p* < 0.01), low CXI (*p* < 0.01), low PMI (*p* < 0.01), low POSSUM mortality rate (*p* < 0.01), cardiovascular disease (*p* = 0.02), chronic renal failure (*p* < 0.01), small bowel obstruction (*p* = 0.02), and ischemia (*p* = 0.03). Multivariate analysis identified age ≥ 75 years (odds ratio [OR] 3.51 [95% confidence interval (CI) 1.13–9.27]; *p* = 0.01), low CXI (OR 2.68 [95% CI 1.20–5.95]; *p* = 0.02) and chronic renal failure (OR 3.18 [95% CI 1.37–7.40]; *p* < 0.01) as independent prognostic factors in‐hospital mortality.

**TABLE 3:** Univariate and multivariate analyses of clinicopathological variables in relation to mortality.

Variables	Univariate analysis		Multivariate analysis	
	Odds ratio (95% CI)	p	Odds ratio (95% CI)	p
Age, ≥ 75	6.72 (2.74–16.45)	< 0.01	3.51 (1.13–9.27)	0.01
Sex, female	1.81 (0.92–3.56)	0.09		
Alb, ≥ 3.3 ng/mL	4.35 (2.09–9.06)	< 0.01	1.43 (0.60–3.37)	0.42
Low CXI	4.82 (2.40–9.70)	< 0.01	2.68 (1.20–5.95)	0.02
Low PMI	4.50 (2.25–9.00)	< 0.01	2.23 (0.99–5.04)	0.06
High NLR	0.75 (0.39–1.47)	0.41		
Malignancy	1.31 (0.48–3.55)	0.60		
Low P‐POSSUM mortality rate	4.89 (2.00–11.97)	< 0.01	2.54 (0.95–6.80)	0.06
Comorbidity				
Cardio vascular disease	2.61 (1.19–5.75)	0.02	1.21 (0.48–3.05)	0.68
Respiratory disease	0.44 (0.06–3.40)	0.43		
Chronic renal failure	6.62 (3.17–13.85)	< 0.01	3.18 (1.37–7.40)	< 0.01
Diabetes	1.95 (0.84–4.53)	0.12		
Cerebral vascular disease	2.03 (0.79–5.22)	0.14		
Postoperative diagnosis				
Small bowel obstruction	0.33 (0.13–0.81)	0.02	0.54 (0.19–1.50)	0.24
Large bowel obstruction	1.93 (0.63–5.96)	0.25		
Perforation of stomach and duodenum	1.07 (0.47–2.43)	0.87		
Perforation of small intestine	1.41 (0.40–4.96)	0.59		
Perforation of colon and rectum	1.33 (0.65–2.70)	0.43		
Ischemia	3.80 (1.15–12.57)	0.03	1.37 (0.34–5.49)	0.66

Abbreviations: Alb, albumin; CXI, cachexia index; NLR, neutrophil–lymphocyte ratio; PMI, psoas muscle index; P‐POSSUM, Portsmouth Physiological and Operative Severity Score for the en Umeration of Mortality and morbidity.

**FIGURE 2:**
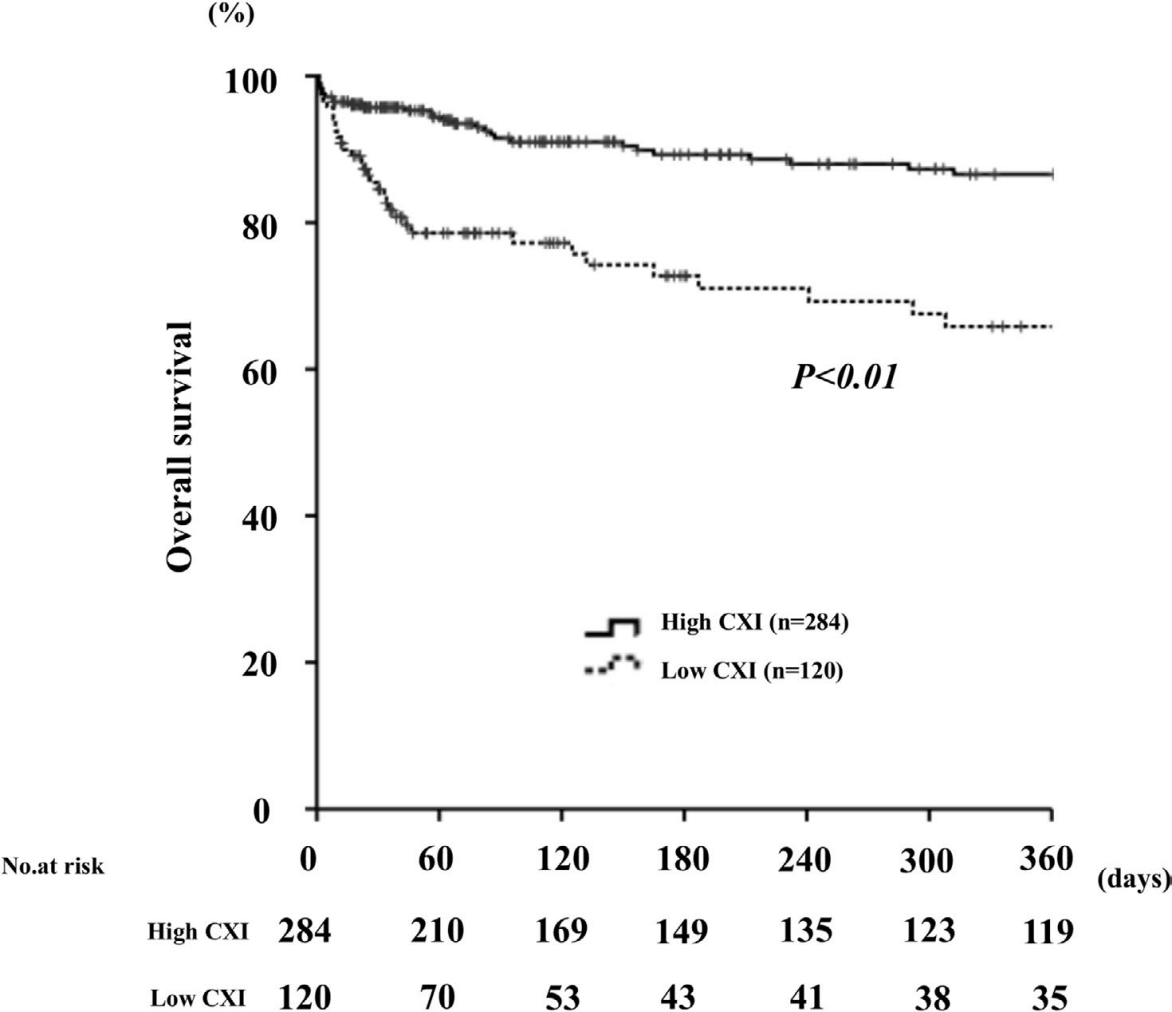
Kaplan–Meier curves showing 1‐year survival for patients with high and low cachexia index.

During the observation period, a total of 93 patients (23%) died. The most common cause of death was sepsis (*n* = 31, 7.7%), followed by cancer (*n* = 25, 6.2%) and pneumonia (*n* = 16, 4.0%). Kaplan–Meier estimates of overall patient survival are presented in Figure 2. Patients with low CXI experienced markedly increased mortality compared to those with high CXI (1‐year mortality, 34.2% vs. 13.4%; *p* < 0.01). In addition, multivariate Cox proportional hazards analysis identified age ≥ 75 years (hazard ratio [HR], 1.99; [95% CI 1.22–3.25]; *p* < 0.01), low CXI (HR 1.59 [95% CI 1.00–2.51]; *p* = 0.04), malignancy (HR 1.93 [95% CI 1.08–3.50]; *p* = 0.03) and chronic renal failure (OR 1.82 [95% CI 1.16–2.86]; *p* < 0.01) as independent predictors of overall survival (Table 4).

**TABLE 4:** Univariate and multivariate analyses of clinicopathological variables in relation to overall survival after emergency laparotomy.

Variables	OS univariate analysis		OS multivariate analysis	
	Hazard ratio (95% CI)	p	Hazard ratio (95% CI)	p
Age, ≥ 75	2.42 (1.57–3.74)	< 0.01	1.99 (1.22–3.25)	< 0.01
Sex, female	1.21 (0.80–1.81)	0.37		
Alb, ≥ 3.3 ng/mL	2.59 (1.70–3.97)	< 0.01	1.41 (0.86–2.29)	0.17
Low CXI	1.95 (1.27–2.97)	< 0.01	1.59 (1.00–2.51)	0.04
Low PMI	2.22 (1.38–3.57)	< 0.01	1.38 (0.83–2.30)	0.21
High NLR	0.92 (0.99–1.01)	1.00		
Malignancy	1.70 (1.02–2.86)	0.04	1.93 (1.08–3.50)	0.03
Low P‐POSSUM mortality rate	2.17 (1.37–3.44)	< 0.01	1.32 (0.80–2.17)	0.28
Comorbidity				
Cardio vascular disease	1.72 (1.02–2.91)	0.04	1.09 (0.63–1.91)	0.76
Respiratory disease	0.37 (0.09–1.49)	0.16		
Chronic renal failure	2.58 (1.72–3.88)	< 0.01	1.82 (1.16–2.86)	< 0.01
Diabetes	1.25 (0.72–2.18)	0.42		
Cerebral vascular disease	1.44 (0.77–2.70)	0.26		
Postoperative diagnosis				
Small bowel obstruction	0.60 (0.37–0.98)	0.04	0.83 (0.49–1.44)	0.51
Large bowel obstruction	1.06 (0.43–2.61)	0.91		
Perforation of stomach and duodenum	0.84 (0.49–1.44)	0.53		
Perforation of small intestine	1.14 (0.50–2.61)	0.76		
Perforation of colon and rectum	1.48 (0.98–2.23)	0.07		
Ischemia	2.03 (0.94–4.38)	0.07		

Abbreviations: Alb, Albumin; CXI, cachexia index; NLR, neutrophil–lymphocyte ratio; PMI, psoas muscle index; P‐POSSUM, Portsmouth Physiological and Operative Severity Score for the en Umeration of Mortality and morbidity.

## 4 Discussion

The present study identified CXI as an independent predictor of in‐hospital mortality and overall survival among a group of patients who underwent EL. Notably, patients with low CXI showed a significantly higher 1‐year mortality rate than those with high CXI. This is the first study to report that a low CXI is closely associated with poor outcomes in patients who undergo EL. These results underscore the importance of a comprehensive assessment of inflammation and physical and nutritional status.

Initially introduced by Jafri et al. [8], in the context of non‐small‐cell lung carcinoma, the CXI has been validated for some malignancies. In a meta‐analysis, Takano et al. [9] demonstrated that the CXI is associated with prognosis in patients diagnosed with gastrointestinal cancers. In the current study, univariate and multivariate analyses revealed that CXI was a more independent predictor of prognosis than PMI, NLR, or Alb alone. Patients with low CXI not only exhibited increased in‐hospital mortality but also higher 1‐year mortality, suggesting that the cytokine response triggered by surgery may initiate a sustained cycle of inflammation and immunosuppression, potentially resulting in prolonged critical illness and subsequent death. The utility of the CXI stems from its capacity to concurrently mirror long‐term physiological reserves and acute systemic stress responses. This dual representation may account for its enhanced prognostic utility in emergency surgical contexts. Although Alb, NLR, and PMI have each been individually reported as prognostic indicators in the emergency surgical setting, our findings suggest that their integration into CXI provides more than the sum of its parts. Rather than simply combining three parameters, CXI captures the interaction between nutritional status, systemic inflammation, and muscular reserve, which often coexist and synergistically influence outcomes in emergency surgical patients. This integrative perspective may explain why CXI demonstrated superior prognostic value compared with each parameter alone. Moreover, conventional scoring systems often require numerous input variables, complex calculations, or specialized software, which can limit their utility in time‐constrained emergencies. In contrast, the CXI is calculated using 3 routinely available parameters, making it a highly feasible and time‐efficient tool for perioperative risk assessment.

Alb is widely recognized as a marker of nutritional status, reflecting visceral protein stores and hepatic synthetic functions. Hypoalbuminemia is commonly observed in critically ill patients and is associated with impaired wound healing, increased risk for postoperative infection, and higher perioperative mortality [14]. In the context of emergency surgery, in which systemic inflammation and catabolism are frequently exacerbated, low Alb level serves as a surrogate for diminished physiological reserves and heightened vulnerability to surgical stress.

NLR is an emerging biomarker of systemic inflammation that reflects the balance between pro‐inflammatory and regulatory neutrophils. High NLR indicates an exaggerated innate immune response and impaired adaptive immunity, which may promote tissue damage, impair healing, and increase the risk for postoperative complications. Several studies have demonstrated that an elevated NLR is associated with poor prognosis in gastrointestinal malignancies and adverse outcomes in both elective and emergency surgeries [15, 16].

Sarcopenia is characterized by progressive and generalized loss of skeletal muscle mass and strength, and reflects general health status [17]. Changes in muscle mass are less prone to short‐term fluctuations and are not influenced by the acute phase of illness. Rather than reflecting the acute severity of the disease, muscle mass indicates overall chronic health status, making it a valuable marker of a patient's general condition before surgery [18]. Currently used methods for assessing sarcopenia include bioelectrical impedance analysis, dual‐energy X‐ray absorptiometry, and CT, all of which have shown strong associations. In EL, a simple and rapid assessment tool is necessary due to urgency; therefore, sarcopenia was evaluated by measuring only the psoas muscle on CT [19]. Several recent reports have suggested that sarcopenia is useful for prognostic assessment before EL. Body et al. [20] reported that sarcopenia was significantly associated with higher mortality in 610 patients who underwent EL. Park et al. [6] demonstrated that after EL, mortality was significantly higher among patients with sarcopenia in the 1‐year in the meta‐analysis (OR 2.8 [95% CI 1.5–5.6]; *p* = 0.002).

Muscle is primarily composed of proteins, accounting for approximately 50% of the total protein in the body. It serves as a nutrient reservoir, distributing amino acids to various organs as part of the body's defense response to physiological stress. However, during severe infections, inflammatory cytokines accelerate proteolysis. Thus, initially having greater muscle mass is advantageous for tissue repair as a defense mechanism, potentially preventing organ failure [21]. Given that preoperative interventions are often limited in EL, these findings highlight the importance of long‐term outpatient efforts to maintain muscle mass and nutritional status. Early identification and nutritional support for at‐risk individuals in routine care may strengthen their physiological reserves before an acute illness. Furthermore, among patients with a low CXI after surgery, tailored postoperative management, such as intensified nutritional therapy, early mobilization, and anti‐inflammatory measures, may help reduce complications and improve recovery. Although direct evidence supporting CXI‐guided interventions is limited, strategies addressing their components have shown benefits in similar high‐risk populations [22, 23].

A systematic and objective preoperative risk assessment is important not only for physicians' decision‐making but also for providing preoperative information to patients and their families. For patients with significantly high‐risk, palliative care may also be considered as a treatment option [24]. Integrating CXI into the preoperative workflow may support individualized treatment goals, particularly for older or frail patients, and help prevent overtreatment in those unlikely to benefit from aggressive intervention [25]. However, a low CXI should not be used as the sole determinant in recommending palliative care. In accordance with recent guidelines recommending comprehensive frailty assessment and shared decision‐making throughout the perioperative pathway for frail patients, CXI should be incorporated as part of a multidisciplinary and ethically grounded assessment framework that considers comorbidities, frailty, functional status, and patient and family preferences [26].

Our study had several limitations, the first of which was its retrospective, single‐center design. This design may have introduced selection bias and unmeasured confounders, which could have influenced the observed associations. Second, the CXI cut‐off value was determined using data from a single institution and, as such, may lack generalizability. Although ROC curve analysis enabled stratification according to sex, the lack of a universally accepted threshold remains a barrier to its broad clinical adoption. Third, while PMA was measured by two experienced surgeons using a standardized protocol and blinded to clinical outcomes, inter‐rater reliability was not formally assessed, leaving the possibility of minor measurement variability. For CXI to be adopted as a reliable prognostic marker in clinical practice, external validation in large‐scale, multicenter, prospective studies is essential to confirm its reproducibility and clinical utility.

In conclusion, the CXI may be a valuable predictor of in‐hospital mortality in patients who undergo EL. Its incorporation into perioperative risk assessment could improve patient stratification and promote personalized clinical care.

## Author Contributions

**Naoko Fukushima:** conceptualization, project administration, writing – original draft, writing – review and editing, visualization, resources. **Takahiro Masuda:** conceptualization, investigation, writing – review and editing, funding acquisition, validation. **Kazuto Tsuboi:** data curation, formal analysis, methodology, writing – review and editing. **Masami Yuda:** data curation, investigation. **Keita Takahashi:** data curation, visualization, validation, software. **Fumiaki Yano:** formal analysis, supervision. **Ken Eto:** writing – review and editing, methodology.

## Ethics Statement

This study was approved by the Ethics Committee of Fuji City General Hospital (296).

## Conflicts of Interest

The authors declare no conflicts of interest.

## Supporting information


**Figure S1:** The optimal cut‐off value of CXI was 1.43 for males (A) and 1.06 for females (B).
