# Supplementary figures and images for: Prognostic Utility of the Preoperative Cachexia Index in Patients Undergoing Emergency Laparotomy
Source: Ann Gastroenterol Surg. 2025 Oct 6;10(2):602–10. doi: 10.1002/ags3.70097 (PMC12962014; doi:10.1002/ags3.70097)

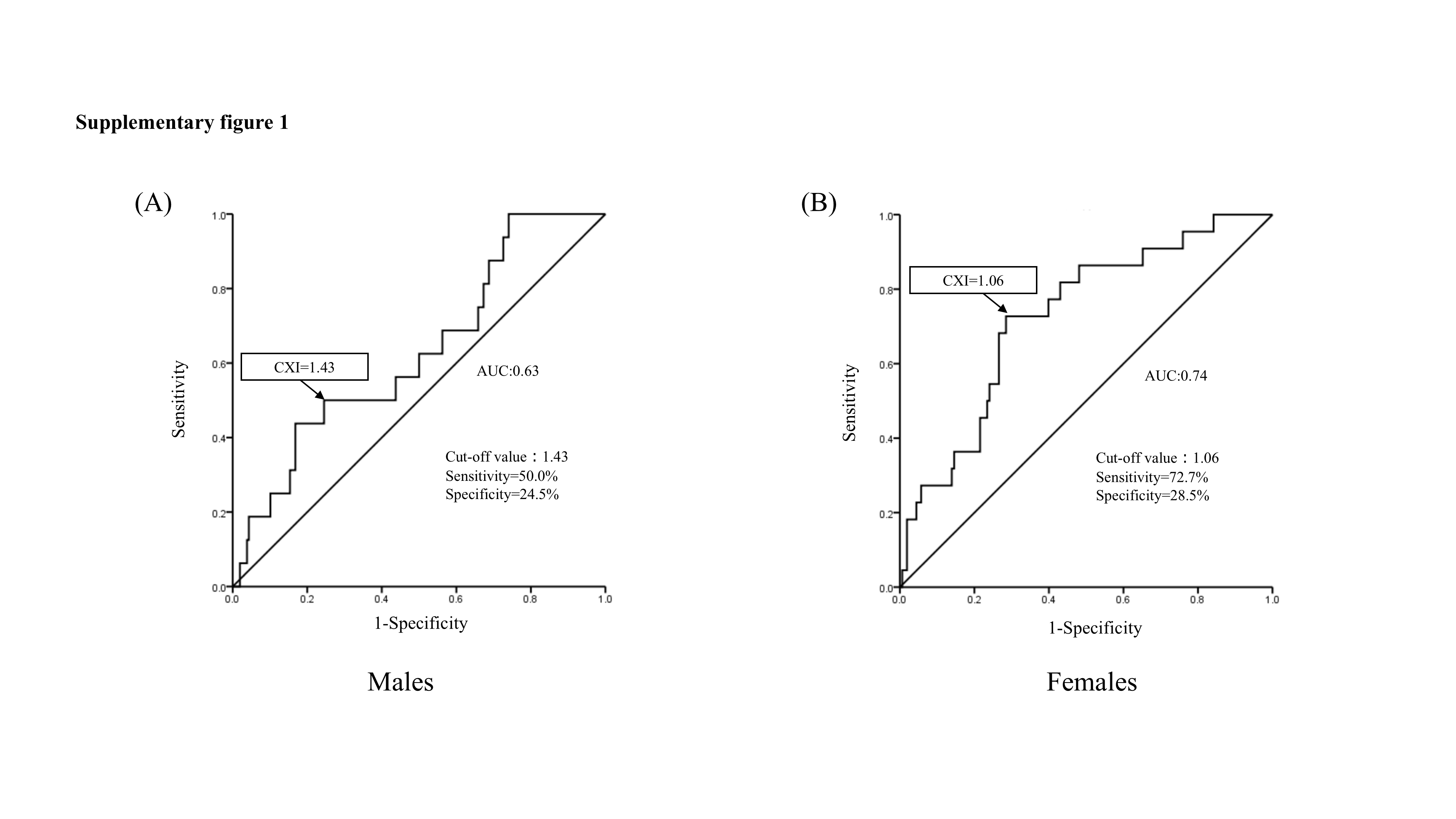

Supplement: Supplementary file 1 — Figure S1: The optimal cut‐off value of CXI was 1.43 for males (A) and 1.06 for females (B). [file AGS3-10-602-s001.tif]
